# Reward-based prioritization and perceptual feature effects on attentional flexibility in working memory

**DOI:** 10.3758/s13414-026-03298-5

**Published:** 2026-06-21

**Authors:** Allison N. Marino, Kelly Cotton, Timothy J. Ricker, Joshua Sandry

**Affiliations:** 1 Psychology Department, Montclair State University, 1 Normal Ave., Montclair, NJ 07043 USA; 2 Department of Neurology, Renaissance School of Medicine, Stony Brook University, Stony Brook, NY USA; 3 Department of Psychology, University South Dakota, Vermillion, SD USA

**Keywords:** Working memory, Focus of attention, Prioritization, Pop-out, Reward

## Abstract

The focus of attention (FoA) is a resource within working memory (WM) assumed to hold limited information in a highly accessible state. Recent evidence suggests the FoA can flexibly adjust to hold early, middle, or terminal memory representations in a highly active state with adequate motivation. When a high-reward item is present, the FoA can be redirected to flexibly adjust and select prioritized items, leading to improved performance. This has been termed the prioritization effect. This finding has been demonstrated across prior investigations that have consistently used the color red to indicate the high-reward stimulus. The salience of the stimulus may alternatively explain the prioritization effect in that the salient item may have an easier time entering WM. Salience could influence memory performance through better encoding. The current study aims to test the effect of feature-based confounds (pop-out effect) on reward-based prioritization by using a probe-recognition task and alternating the color of the to-be-prioritized item. Four colors (red, green, blue, magenta) were used, and the color assigned to a higher point-value was randomized between subjects. Our results showed that the prioritization effect was still evident even when salience (pop-out) was controlled. Regardless of the color used there was a performance benefit for the prioritized list position. Comparisons with prior studies indicate that visual pop-out, while not necessary for prioritization, may help facilitate stronger reward effects. Raw trial-level data, statistical analysis, and task code are available in the Open Science Framework (OSF) repository (https://osf.io/xsc34/overview). The experiment was not preregistered.

## Introduction

When coordinating between multiple responsibilities, such as remembering to send an important email, picking up groceries, or responding to a text message, people naturally focus on the task with the highest perceived value. This tendency to focus attention on more rewarding or consequential information is well documented and termed the prioritization effect. Prioritized or high-value information receives a performance benefit across visual, verbal, auditory, and tactile modalities (see Allen et al., 2024, for a review). Importantly, this advantage typically comes with a corresponding performance cost to non-prioritized items, reflecting an attentional resource trade-off across memory items (Atkinson et al., 2018, 2024; Sandry et al., 2014, 2020; Sandry & Ricker, 2020; Ricker et al., 2026). This trade-off demonstrates the ability to flexibly allocate limited attentional resources towards specific information when there is a benefit to do so.

This flexibility is especially evident within working memory (WM), the capacity-limited cognitive system that integrates attention and memory (Cowan, 2001). Within WM, the focus of attention (FoA) is capacity limited and assumed to maintain information in a highly accessible state that guides current thoughts and actions (Cowan et al., 2024). The prioritization effect is observed when certain to-be-remembered items are associated with a high reward. Those high-reward items are maintained within the FoA in a more accessible state than low-reward items within the same set. Reward-based manipulations robustly elicit the prioritization effect across various task designs (Allen & Ueno, 2018; Atkinson et al., 2024; Hitch et al., 2018, 2020; Hu et al., 2014, 2016, 2023; Labaronne et al., 2024; Ricker et al., 2026; Sandry & Ricker, 2020; Sandry et al., 2014, 2020).

In prior investigations, we have utilized a probe-recognition paradigm in which three to-be-remembered, sequentially presented stimuli are shown with a high and low point value manipulation. On 75% of trials, one item displayed in red was high reward and worth 25 points while other items presented in black were low reward and worth 3 points. High-reward items were consistently responded to faster or more accurately compared to low-reward items (Sandry & Ricker, 2020; Sandry et al., 2014, 2020). The prioritization effect is likely not due to distinctiveness of encoding (Sandry & Ricker, 2020) and is distinct from the recency effect (Ricker et al., 2026). It remains possible and untested that the prioritization effect and resource trade-off is facilitated by the single red pop-out item in combination with top-down behavioral goals, boosting performance above and beyond reward value alone.

Although past work has attempted to rule out color-based confounds, the issue of separating perceptual features from top-down goals remains unresolved. Sandry and Ricker (2020) ruled out the von Restorff isolation effect, which suggests that items with distinct or salient features are better remembered than groups of similar or non-salient items. To test this, a between-subjects manipulation contrasted high-reward and equal-reward conditions. In the high-reward condition, red-list positions were worth more points than black-list positions and the standard prioritization effect was observed. In the equal-reward condition, red- and black-list items were worth equal points, and under this configuration, the single red item did not produce any performance advantages over black-list items. That is, there was no prioritization effect without reward, suggesting that color features do not drive memory benefits observed in the high-reward condition. Importantly, in reward-based versions of this specific task, the high-reward item is always a red singleton among a set of otherwise black items. Therefore, the visual popout is always aligned with the participant’s behavioral goals to prioritize that high-reward item. It may be that this visual pop-out is necessary for, or greatly facilitates, online item prioritization during rapid item presentation. Without this visual pop-out, online item prioritization may be greatly diminished or abolished entirely. Thus, the potential interaction between visual salience and reward-based prioritization has not been completely isolated. If this interaction is necessary to observe prioritization effects, it would strongly challenge existing flexible attention theories across prioritization paradigms.

Prioritization effects have been demonstrated in paradigms that do not vary perceptual salience (Atkinson et al., 2024; Hitch et al., 2018, 2020; Hu et al., 2014, 2016, 2023), suggesting that perceptual distinctiveness is not a necessary factor for prioritization benefits to emerge. However, in this line of research prioritization cues are given before the memory items are presented. This may provide individuals with a brief period of time to prepare attention-allocation strategies. In other investigations, including those presented here, prioritization is conducted online with the prioritization cue being presented endogenously as part of the memory item (Cinar et al., 2025; Sandry & Ricker, 2020; Sandry et al., 2014, 2020). In these paradigms high reward is always associated with perceptual pop-out, leaving open the possibility of salience modulating effects observed in online prioritization tasks.

### Color and salience

This salience-plus-reward alternative is especially important to evaluate when considering current literature describing color effects in memory and attention. Specifically, the color of stimuli can improve encoding, storage, and retrieval mechanisms within memory (see Dzulkifli & Mustafar, 2013, for a review). Participants can recognize the color of stimuli better than its shape (Pan, 2010) and respond faster to colored versus black and white stimuli (Vernon & Lloyd-Jones, 2003), suggesting that color can increase attentional capture leading to performance benefits. Red has been shown to enhance detail-oriented performance (Mehta & Zhu, 2009), implying that red may be especially beneficial for goal-oriented tasks and behaviors. Behavioral studies in selective attention have presented evidence that color captures attention in WM both voluntarily and involuntarily (see Folk, 2015, for a review).

Some evidence supports inflexible encoding, where salient stimuli are assumed to enter WM in a more involuntary fashion (Harris & Pashler, 2004; Roer & Cowan, 2021). Attention is automatically drawn to the item with the greatest salience regardless of relevance (Constant & Liesefeld, 2023). Other evidence suggests flexible encoding, where individuals can suppress salient information that is not task relevant (Cowan et al., 2024; Folk et al., 1992; Gaspelin et al., 2015, 2017; Gaspelin & Luck, 2018; Lorenc et al., 2021) and features can act as a facilitator depending on behavioral goals. For instance, Folk et al. (1992) demonstrated that attentional capture by a salient color singleton occurs when the color matches top-down attentional control goals. Electrophysiological evidence also supports this whereby attentional modulations from target pop-outs (Eimer et al., 2010; Luck & Hillyard, 1994) and luminance changes (Johannes et al., 1995) show an increase in amplitude and latency in various early and late-stage event-related potential components. These disparate findings suggest that attentional capture by features may be partially or fully facilitating top-down behavior goals. When a salient feature such as color aligns with task goals, this may amplify attention and memory performance.

Reward-based prioritization effects observed in prior work may in part be driven by feature-based attentional capture rather than the reward value itself. If the prioritization effect is reduced or not observed under conditions when reward is manipulated with no visual popout, this may indicate that features and their alignment with task goals are needed to enhance performance benefits. Alternatively, if prioritization effects remain even when no visual popout is included, this would provide support for the flexible attention theory of prioritization. Understanding this distinction is necessary to accurately interpret prioritization effects and in determining whether and how they truly reflect flexible resource allocation within WM or instead arise from perceptually driven attentional capture.

### Present experiment

In the present experiment we replicated Experiment 1 of Sandry et al. (2014), a probe-recognition paradigm utilizing letter stimuli as memory items. On some trials, one list position is associated with a higher point value to induce prioritization. In contrast to Sandry et al., each letter in the current experiment was presented in a different color selected from a set of four colors (red, green, blue, magenta) with matched luminance. One color was used to assign high reward while the remaining three colors indicated low-reward values, thereby controlling for low-level perceptual salience across items. This manipulation allowed us to test the magnitude of the prioritization effect based exclusively on reward value rather than reward value interacting with visual popout. To evaluate this, we contrasted the inflexible encoding theory against the flexible encoding theory.

The flexible encoding theory predicts that encoding resources can be flexibly and voluntarily distributed to goal-relevant stimuli and thus high-reward items will receive more resource allocation compared to low-reward items, even when perceptual salience is constant. If the prioritization effect is observed under equal perceptual salience, this would support a flexible, prioritization mechanism, consistent with previous findings. In contrast, the inflexible encoding theory predicts that attention is captured automatically by perceptually salient features, thus when visual popout is eliminated, there should be no difference in high- and low-reward performance. If the prioritization effect is not observed under equal perceptual salience, this would suggest that encoding is equally distributed across items even when top-down behavioral goals are present. Testing these flexibility theories of capture into WM is important for understanding the magnitude of reward-based prioritization within WM, a process constantly in use with everyday behaviors and goals. This study will also help establish the validity and replicability of prior investigations as well as add to the robust nature of the prioritization effect for sequentially presented information.

## Method

Data for the present experiment are available at https://osf.io/xsc34/overview. The experiment and analyses were not preregistered.

### Participants

Ethical approval for this study was obtained from the Institutional Review Board at Montclair State University. We recruited 149 undergraduates (108 female and 41 male) to participate in this study for partial course credit. In prior investigations using this paradigm and a between-subjects design, we identified a reliable effect with 30 participants per condition (Sandry & Ricker, 2020). We recruited a minimum of 30 participants for each of our four between-participants conditions (high-reward color), for a combined minimum recruitment of 120 participants. Actual recruitment was slightly above this target in order to account for likely performance-based participant exclusions. The mean age was 21 years (*SD* = 5.19). All participants reported no color deficiency, which was verified using an Ishihara test (https://www.colorblindnesstest.org/ishihara-test/).

### Materials and design

**Fig. 1:**
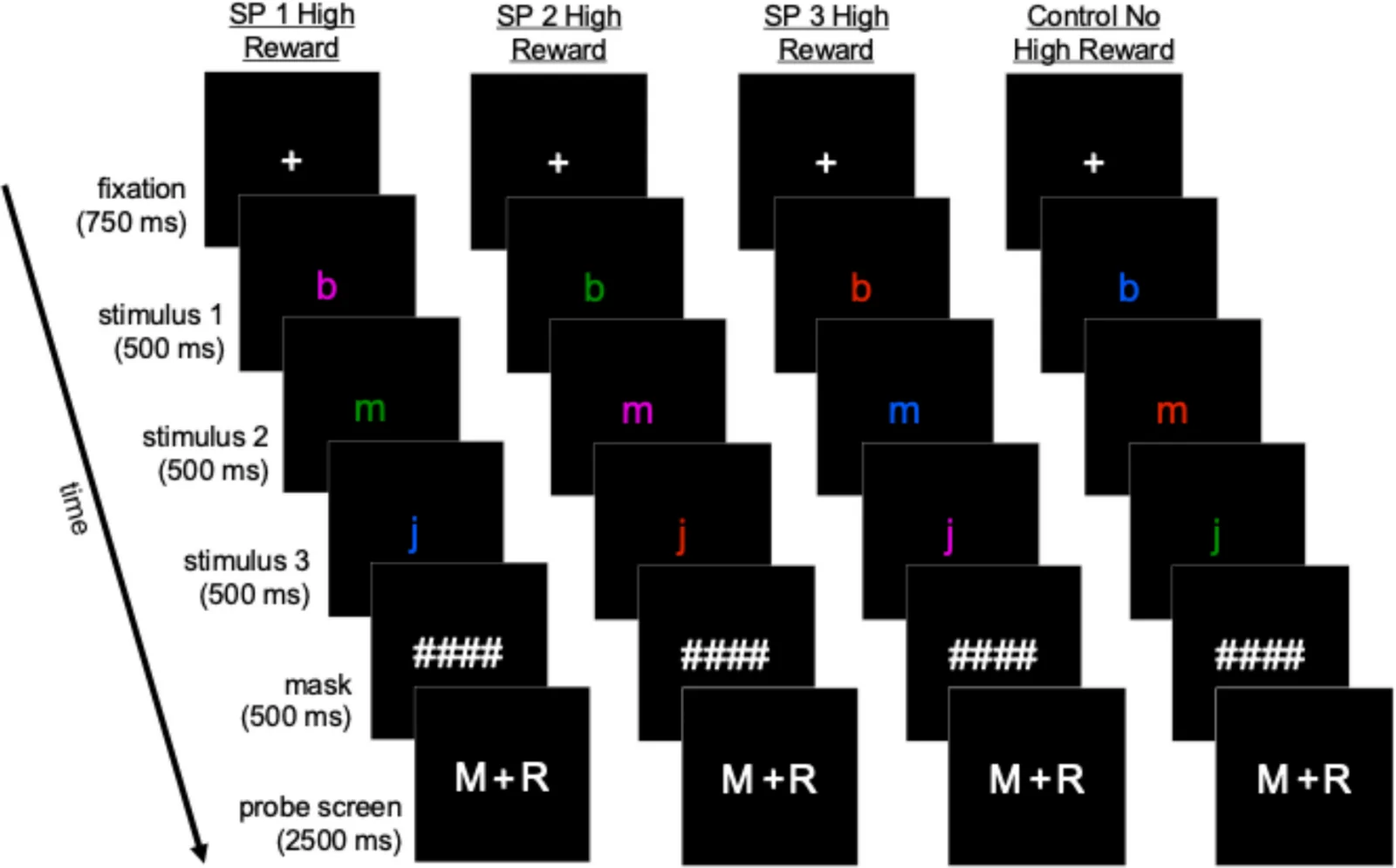
Procedure used in the current study. Fixation cross (500 ms), followed by three sequentially presented to-be-remembered letters (500 ms each), and a mask (500 ms), followed by a probe screen (2,500 ms). Participants were instructed to indicate whether the letter that was shown in the list on a given trial appeared on the left or right of the probe screen using the “D” and “K” keys, respectively. In this Figure, magenta is shown as the color used to assign high reward. SP = serial position

The experiment was run using PsychoPy versions 2023.2.3 and 2025.1.1 on Mac computers with a 24-in. monitor with a resolution of 4,480 x 2,520 pixels and a set distance of 65 cm. The design replicated Experiment 1 of Sandry et al. (2014) but with stimulus colors changed. Instead of using red for high reward and black for low reward, we modified the task to include four possible colors (red, green, blue, magenta). These colors were randomized across list positions and the color signaling high reward was counterbalanced across participants. Each trial consisted of three sequentially presented letters randomly selected without replacement from the set of B, F, G, H, J, L, M, Q, R, T, and Y. These letters were all sized 1.0º in visual angle in Open Sans font presented on a black background and varied in color presentation of red, green, blue, or magenta. Colors were selected from CIELAB space to maintain equal luminance values (Red: L* = 50, a* = 70, b* = 70, Green: L* = 50, a* = −70, b* = 70, Blue: L* = 50, a* = 55, b* = −120, Magenta: L* = 50, a* = 100, b* = −60) (see Fig. 1). To-be-remembered stimulus letters were presented in the same lowercase point size and font. Test stimuli were presented in uppercase to minimize similarity recognition. The design was a 4 Prioritization Position (high-reward stimulus in the First, Middle, or Last list position or only low-reward stimuli in each list position [Control]) X 3 Probe Position (First, Middle, or Last) within-subjects design with High Reward Color (red, green, blue, or magenta) as a between-subjects factor resulting in a 4 X 3 X 4 mixed factorial design.

### Procedure

The procedure is visualized in Fig. 1. The experiment was structured as a verbal working memory task where participants could earn points for a correct response and lose points for an incorrect response. Participants viewed three sequentially presented letters worth 3 points each and were asked to identify which letter had appeared in the list from a two-alternative forced-choice (2AFC) screen that contained one letter from the three-item list and one not from the list. Participants were instructed to focus on maintaining high accuracy and point accumulation. On 75% of trials, one colored letter (counterbalanced between participants) was worth 25 points. Each serial position had an equal probability of being presented in the high-reward color and, separately, an equal probability of being probed.

Each trial began with a fixation cross (750 ms) that remained present over the course of the trial. Then, three to-be-remembered letters were presented sequentially (500 ms each), followed by a mask (######: 500 ms). A probe screen (2,500 ms) followed and presented one letter from the list and one letter that was not shown on that trial. One letter was on the left and one letter was on the right of the central fixation cross. Participants responded by key press to indicate which letter was from the list, with “D” for left or “K” for right. The correct answer had an equal probability of appearing on the left or right side of the response screen and appeared at each location an equal number of times. At test, responses had to be made within 2,500 ms. If a response was not made within this time frame, the probe screen would time out and be marked as incorrect and proceed to the next trial. After a response the feedback screen appeared showing whether the answer was correct, the current point accumulation, and block accuracy (1,500 ms).

The task began with 12 practice trials, followed by 288 experimental trials. Participants were given instructions and practice trials for the task without the presence of a high-reward letter before beginning experimental trials. After completion of practice trials, participants were informed through an instruction screen that the high-reward color indicated higher point value. After every 48 trials, participants took a 30-s break, during which a screen indicating their current point accumulation total and overall accuracy were displayed. Participants were instructed to respond as quickly and accurately as possible. The experiment lasted approximately 30 min, and the experimenter monitored participants throughout the task.

## Results

### Data preprocessing and statistics

Data preprocessing followed the approach used in our previous studies with this paradigm (Ricker et al., 2026; Sandry & Ricker, 2020; Sandry et al., 2014, 2020). Participants were excluded for mean accuracies below chance levels of performance (*n* = 1) or ≤ 2 standard deviations below group-level mean (*n* = 5) as their attention and effort could not be confirmed. Analyses on response time (RT) were conducted on accurate trials only. Participants were also excluded if their mean RT met or exceeded 2 standard deviations shorter/longer than the group-level mean (*n* = 8). After exclusions, the final sample included 135 participants (High-Reward group Magenta: *n* = 37, Blue: *n* = 32, Green: *n* = 33, Red: *n* = 33) for analyses. Participant-level RT outliers were operationalized as RTs shorter than 300 ms or longer than 3 standard deviations above the participant’s mean in each experimental condition (Ricker et al., 2026; Sandry & Ricker, 2020; Sandry et al., 2014, 2020). This resulted in removal of 1.27% of trials. The results were analyzed using a 4 x 3 x 4 (prioritization position x probe position x high reward color) mixed-measures ANOVA with a Greenhouse-Geiser correction for sphericity. To foreshadow, accuracy was near ceiling and we primarily interpret RTs.

### Accuracy

**Fig. 2:**
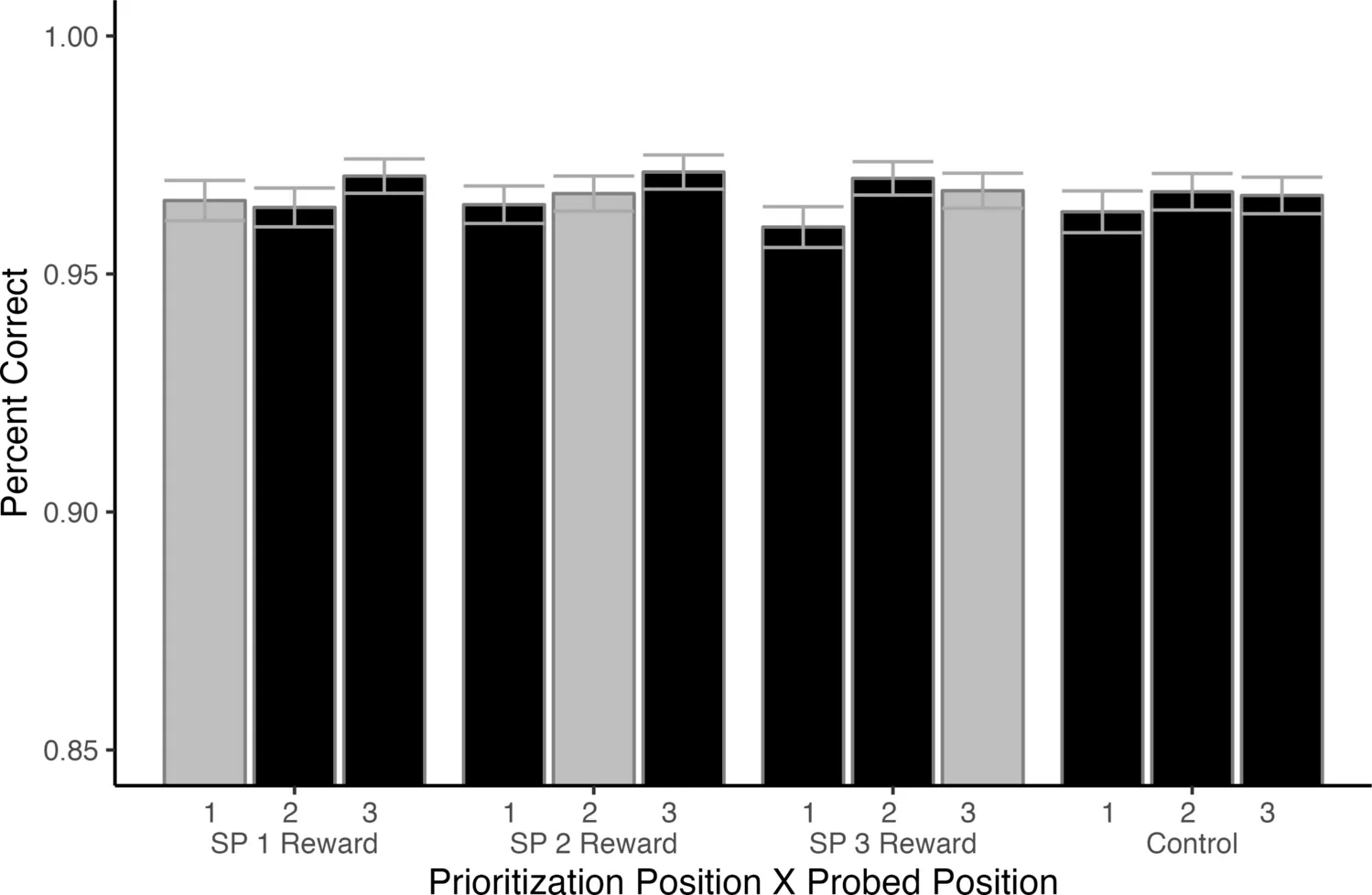
Mean accuracy for Prioritization Position and Probe Position. The gray bars represent prioritized high-reward list positions (across all high-reward colors), and the black bars represent low-reward list positions (low-reward colors were randomized). Error bars represent standard errors of the means. SP = serial position

In line with Sandry et al. (2014), accuracy approached ceiling (*M* = 97%) limiting any meaningful interpretation of accuracy (Fig. 2). There was no main effect of high-reward color, *F*(3, 131) = 0.06, *p* = 0.982. Additionally, there was no interaction between the high-reward color and prioritization position, *F*(8.80, 384.22) = 0.72, *p* = 0.685, or probed position, *F*(5.94, 259.35) = 0.51, *p* = 0.798. Further, there was no significant three-way interaction, *F*(17, 742.50) = 0.90, *p* = 0.574. The main effect of prioritization position was not significant, *F*(2.93, 384.22) = 0.26, *p* = 0.848, the main effect of probed position was not significant, *F*(1.98, 259.35) = 2.94, *p* = 0.055, *η*^*2*^*G* = 0.003, and the interaction between prioritization position and probed position was not significant, *F*(5.67, 742.50) = 0.69, *p* = 0.65.

### Response time

**Fig. 3:**
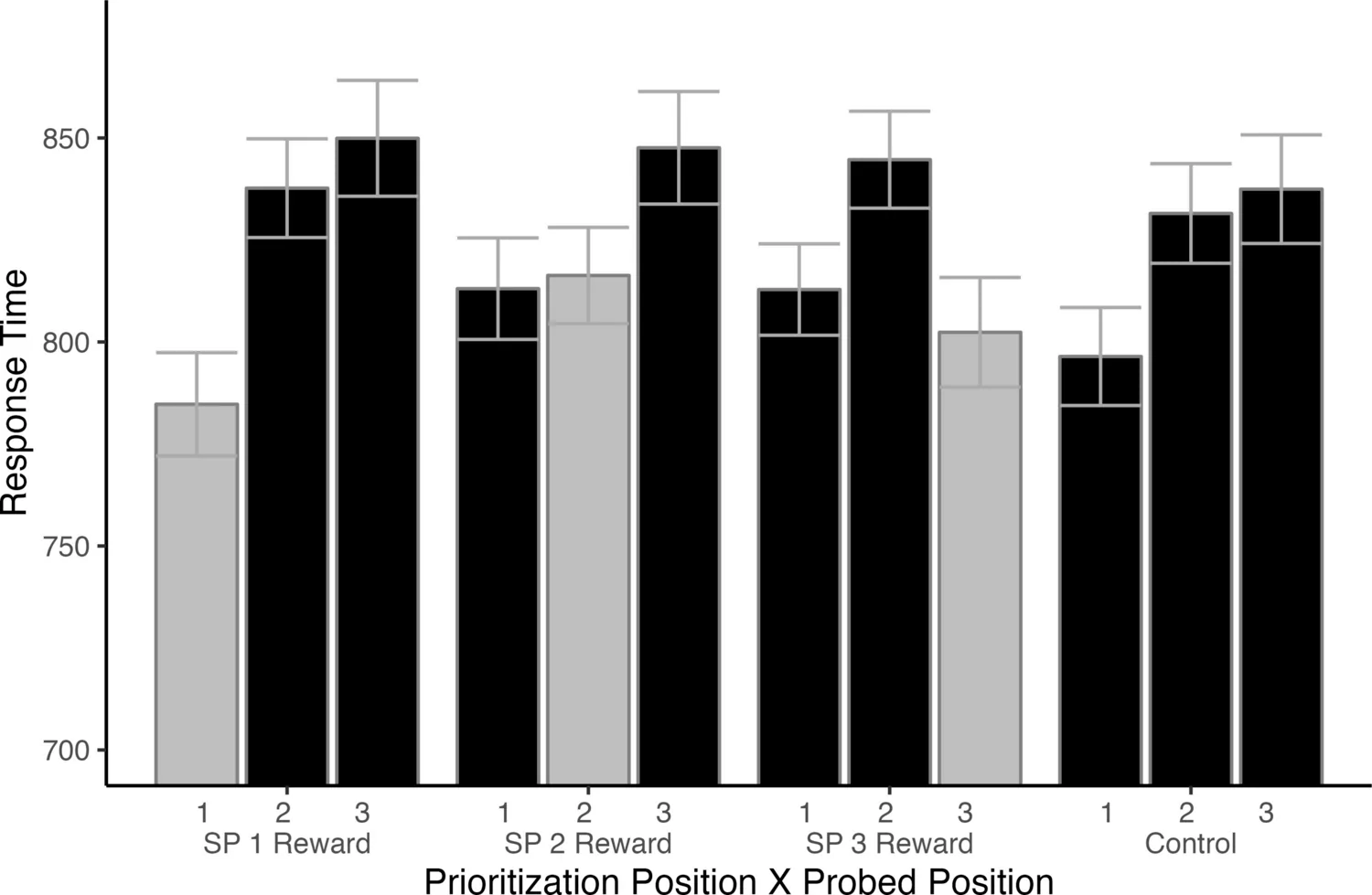
Mean response times (in milliseconds) for Prioritization Position and Probe Position. The gray bars represent prioritized high-reward list positions (across all high reward colors), and the black bars represent low-reward list positions (low-reward colors were randomized). Error bars represent standard errors of the means. SP = serial position

Mean RTs collapsed across all high-reward color conditions are visualized in Fig. 3 for each prioritization and serial position. There was no difference in prioritization as a function of the four colors used in the task. This was supported by the following statistical patterns when including the high-reward color condition in the model. There was no main effect of high-reward color, *F*(3, 131) = 1.05, *p* = 0.373. Additionally, there was no interaction between the high-reward color and prioritization position, *F*(8.61, 376.08) = 1.43, *p* = 0.177 or probed position, *F*(5.06, 220.87) = 1.00, *p* = 0.422. Further, there was no significant three-way interaction, *F*(16.19, 707) = 0.98, *p* = 0.482. Given that there was no effect of color, all further analyses of prioritization and probed positions were conducted collapsing across high-reward color to explore whether the prioritization effect aligned with prior research. The main effect of prioritization position was not significant, *F*(2.87, 376.08) = 0.64, *p* = 0.584. The main effect of probed position was significant, *F*(1.69, 220.87) = 15.04, *p* < 0.001, *η*^*2*^*G* = 0.011, as was the interaction between prioritization position and probed position, *F*(5.40, 707) = 12.71, *p* < 0.001, *η*^*2*^*G* = 0.01. Follow-up comparisons are presented in Table 1. Briefly, patterns supporting the prioritization effect (Table 1, row A) replicated Sandry et al.’s (2014) Experiment 1. Comparisons of prioritization position and recency effects indicated that participants responded faster to prioritized items in the first and middle serial positions than to non-prioritized items in the last serial position (Table 1, row B). In contrast to prior work, only weak evidence of a resource trade-off was observed (Table 1, row C).

**Table 1:** *t-*Test comparisons for prioritization and serial position. All comparisons are labelled as Prioritization position: Probed position. Row A comparisons are between the prioritized position and control at the same list position. Row B comparisons are between the prioritized position and recency effects in the same condition. Row C comparisons are between the unprioritized first, middle, or last probe position from the control condition and the unprioritized first, middle, or last list position when the first or middle position was prioritized

	Prioritization position: Probed position	Response time			Accuracy		
		*t*(134)	*p*	*d*	*t*(134)	*p*	*d*
A	sp1 reward: sp1 vs. control: sp1	1.55	.12	.13	−0.5	.62	.04
	sp2 reward: sp2 vs. control: sp2	2.09	.04	.18	0.09	.93	-.01
	sp3 reward: sp3 vs. control: sp3	4.19	.001	.36	−0.24	.81	.02
B	sp1 reward: sp1 vs. sp3	6.10	.001	.52	1.05	.30	-.09
	sp2 reward: sp2 vs. sp3	3.33	.001	.29	1.03	.31	-.09
C	sp1 reward: sp3 vs. control: sp3	1.55	.12	.13	−0.82	.41	.07
	sp2 reward: sp3 vs. control: sp3	1.53	.13	.13	−1.33	.19	.11
	sp1 reward: sp2 vs. control: sp2	0.80	.42	.06	1.39	.17	.12
	sp2 reward: sp1 vs. control: sp1	2.34	.02	.20	1.69	.09	.14

## Discussion

In this study, we sought to eliminate visual pop-out effects to determine whether reward-based prioritization operates separately from perceptual salience while also replicating attentional allocation changes within the FoA. The findings suggest that reward-based prioritization modulates performance independently of perceptual features, providing support for the flexible encoding theory. RT results showed no differences across the four prioritization colors, while the significant effect for prioritized position relative to control conditions indicated that participants flexibly allocated their attentional resources to prioritized list positions when motivated by reward without the presence of a stimulus driving automatic attention capture. Prioritized positions also overcame recency effects. That is, participants responded faster to high-reward items in the first or middle list positions than low-reward items in the last list position (see Allen et al., 2014). In contrast to past work, we observed a weak attentional resource trade-off and we elaborate on this inconsistency below.

### Salience

Although the prioritization effect was supported, comparisons with earlier studies of prioritization reveal a few notable differences. Sandry et al. (2014, Experiment 1a), which used the same task and procedure with visual pop-out, reported larger effect sizes across each prioritized position compared to control conditions (sp1: Cohen’s *d* = 0.59, sp2: *d* = 0.38, sp3: *d* = 0.68; Current study: sp1: *d* = 0.13, sp2: *d* = 0.18, sp3: *d* = 0.36). Additionally, this past investigation reported a significant benefit for the prioritized position at the first serial position. This was not statistically supported in the current study, although the mean patterns suggest an effect is present (see Fig. 3). This discrepancy may be attributable to the automatic capture of target stimuli in this earlier work, which likely strengthened encoding of high-value items, making them more resistant to forgetting due to their distinctiveness (Smith & Mulligan, 2018). Consistent with this interpretation, pop-out or salient targets in visual search tasks are detected more rapidly (Eimer et al., 2010; Geyer et al., 2010; Nothdurft, 2006). This is likely due to pre-attentive neural mechanisms related to bottom-up salience that operate prior to controlled awareness (Hsieh et al., 2011; Li, 2002) leading to feature-detection to enhance task performance. Although perceptual pop-out was effectively eliminated in the present study, the reduced effects observed here suggest that perceptual features may still facilitate or augment online item prioritization during rapid item presentation, thereby amplifying performance benefits. Future work should directly contrast a salient reward popout condition (e.g., Sandry et al., 2014) versus a non-salient reward condition (e.g., the present procedure) in the same experiment. This will provide insight into the magnitude of reward differences when paired with and without visual salience. While the present findings provide converging evidence in support of prioritization, it is likely that there is a small influence from mechanisms related to salience.

The relatively weaker effects of prioritization observed here are consistent with studies that have employed endogenous rewards, strategy use, or retro-cueing, which often show more variable outcomes or modest benefits (Allen & Atkinson, 2021; Allen & Ueno, 2018; Atkinson et al., 2018; Klyszejko et al., 2014; Makovoski & Jiang, 2007; Zhang & Luck, 2011; Zhang & Lewis-Peacock, 2023). For instance, Klyszejko et al. (2014) manipulated priority through monetary value, reporting improved precision in visual WM performance, while Zhang and Luck (2011) similarly implemented monetary incentives to improve precision but reported a null effect. Cueing studies utilizing prioritization of multiple items with an equal probability of being tested (Allen & Ueno, 2018) and studies using highly predictive cues (Makovoski & Jiang, 2007) conflict in outcomes of single versus multi-item limits to the distribution of attentional resources for memory enhancements. Taken together, these studies suggest that task design, reward context, and implementation details can differentially impact performance benefits within working memory. While the present study replicated the main patterns of prior work on prioritization effects, design differences indicate that visual popout can have important effects. It may be that eliminating the salience of the prioritized item requires participants to first identify which item carries the high reward, shifting reliance from low effort or automatic processing/capture to more effortful attentional selection. This shift may reduce the benefits of flexible prioritization under brief stimulus presentation, as the prioritized item no longer has a perceptual advantage. Ultimately, this finding suggests that visual pop-out may be necessary to produce the strongest reward effects to guide attention in line with task goals during brief stimulus presentation.

### Strategy use

Opportunities for rehearsal may have introduced additional variability. In Sandry et al. (2014), letter stimuli were used as memory items and were tested with and without articulatory suppression. Comparisons between these two experiments revealed differences attributable to the presence of the suppression requirement. Mainly, a reduction in accuracy was observed, likely reflecting increased cognitive load (Soto & Humphreys, 2008). RTs for the prioritization effect did not differ substantially across experiments. However, support for the resource trade-off model was stronger with articulatory suppression, reflected in greater performance costs for non-prioritized probed items in Sandry et al. (2014) than in the present work. This difference suggests that suppression amplifies the impact of resource allocation by limiting the use of alternative strategies such as rehearsal or by increasing task difficulty (Ricker et al., 2026). In the present study, articulatory suppression was not implemented and thus may have allowed verbal rehearsal of letter stimuli to facilitate encoding independently of prioritization. The use of relatively simple and familiar letters as memory items coupled with the elimination of visual pop-out may have led participants to rely less on reward-based allocation and more on other memory strategies, reducing the advantage of prioritization and instead focusing on overall task goals. Allocating limited attentional resources across all memory items rather than selectively prioritizing a single item may place fewer demands on strategic control as all items are maintained in an active state rather than directing a single item into the FoA to enhance later recall. This explanation is also congruent with a moderating effect of task demands on prioritization.

### Resource trade-off

Accuracy in the present paradigm was especially high, suggesting that the task may have been relatively easy or that the stimulus materials were not complex, limiting processing demands which may also have influenced prioritization patterns. In past research we suggested that stimulus complexity may moderate prioritization effects in WM with more complex stimuli leading to stronger effects (Sandry et al., 2020). When contrasting across studies using this paradigm, results for more complex stimuli such as shapes, unfamiliar characters (Ricker et al., 2026; Sandry & Ricker, 2020) or semantically meaningful information (Sandry et al., 2020) show stronger retrieval speed effects for prioritization. On the other hand, less complex stimuli such as spatial directions (Sandry & Ricker, 2020) and letters (Sandry et al., 2014) show weaker effects. As stimuli become more challenging to store, accuracy decreases but the prioritization and recency effect become more pronounced (Ricker & Sandry, 2025). More complex stimuli may require increased attentional resources leading to a difficult to overcome recency advantage (Sandry et al., 2020). When task demands increase, participants may strategically allocate attentional control by focusing on fewer items, prioritizing high-value or easier to remember (recent) stimuli.

While the complexity hypothesis has not undergone direct experimental testing, prioritization effects may be constrained by boundary conditions that depend on stimulus sets. In the present study, we used highly familiar, easy to rehearse letters, overall causing task demands to be low. It is possible with a more complex stimulus set and with visual pop-out removed, the prioritization effect will be more effective, showing stronger performance benefits across list positions and simultaneously decrease accuracy, which would help eliminate the present ceiling effect. With greater variability in accuracy data, support for a resource trade-off model may be more readily observed and indicate meaningful differences in accuracy in addition to RT when controlling for perceptual features. This question should be examined in future research to test how the prioritization effect changes and when the resource trade-off occurs. Recent computational modeling findings provide strong support for a resource trade-off model (Ricker et al. 2026). The subtle influence of salience observed herein may attenuate when task demands are more challenging or stimuli are more complex, intensifying competition within WM and making prioritization more consequential.

These results have important implications for future physiological research aimed at identifying the neural mechanisms underlying this benefit which have yet to be disentangled. Specifically, it remains unclear whether prioritization effects primarily reflect enhanced encoding processes or later motor preparation mechanisms and how task demands and salience may differentially interact across these processes. In Ricker et al. (2026), drift diffusion modelling was used with this paradigm utilizing complex unfamiliar characters as the stimulus set. Results on non-decision times of prioritized items indicated a strong resource trade-off pattern, suggesting enhancements of perceptual or motor processing. Specifically, these results may reflect enhancement of perceptual processing of the probe or improved motor preparation at the time of test. The present findings contribute to this line of work by demonstrating prioritization effects in the absence of perceptual confounds, providing a clearer foundation for isolating temporal and neural dynamics of reward-based prioritization.

## Limitations

A few limitations of the current study should be noted. Although perceptual distinctiveness was intentionally eliminated to isolate top-down reward-based prioritization, the design choice may have reduced the overall magnitude of prioritization effects. In prior investigations (Ricker et al., 2026; Sandry & Ricker, 2020; Sandry et al., 2014, 2020) red was used to denote priority leading to strong effects for high-reward (red) list positions and an attentional resource trade-off between high-reward and low-reward items. Studies on color and feature effects have indicated that colored stimuli lead to faster responses versus black and white stimuli (Vernon & Lloyd-Jones, 2003) and that features can act as facilitators depending on behavioral goals (Folk et al., 1992). It may be that perceptual features are naturally directing attentional resources. Removing this factor could have made it more difficult for participants to reliably implement prioritization strategies, particularly at early serial positions (primacy) where information is not as easily accessible compared to more recent serial positions.

Additionally, the use of letters as stimuli could have contributed to verbal encoding and rehearsal strategies which possibly obscured reward-based effects. Participants may have relied on rehearsal strategies leading to more easily encoded memory representations that could lead to less reliance on prioritization mechanisms and more on strategy use to combat the lack of perceptual distinctiveness. Future work using difficult to rehearse stimuli may provide a converging test of reward-based prioritization in the absence of perceptual cues.

## Conclusion

In this study, we tested whether reward-based prioritization persists when perceptual pop-out is eliminated. The present findings address this question by demonstrating that items maintained in the FoA can be prioritized with a high reward in the absence of perceptual distinctiveness. However, perceptual features that align with task goals are observed to amplify performance benefits. Top-down goals paired with perceptual features leads to greater effects than top-down goals alone. By eliminating perceptual cues in this task, the current study isolated the contribution of reward value and largely replicated reward-based prioritization effects reported in prior work. The results support a flexible encoding theory of prioritization but diverge from earlier findings: effect sizes were weaker overall and minimal evidence was found towards a resource trade-off model, but this may be due to stimulus characteristics. Together, these results indicate that reward-based prioritization can operate independently of salience, while perceptual distinctiveness is seen to strengthen or magnify its impact. This dissociation provides a clearer foundation for future research aimed at disentangling the neural underpinnings of prioritization in working memory.

## Data Availability

All data and code are available on the Open Science Framework at: https://osf.io/xsc34/overview

## Declarations

### Preregistration

The study was not preregistered.
